# Diabetes-related stress in older adults with type 2 diabetes and chronic complication: Multiple effects of social-ecological support on self-management behavior

**DOI:** 10.1097/MD.0000000000037951

**Published:** 2024-04-26

**Authors:** Xiao Sun, Yan Shi, Xue Wang, Rongrong Zhou, Wei Deng

**Affiliations:** a Nursing Department, Shanghai Fourth People’s Hospital, School of Medicine, Tongji University, Shanghai, China; b Department of Nursing, Tenth People’s Hospital, School of Medicine, Tongji University, Shanghai, China; c Department of Emergency, Tenth People’s Hospital, School of Medicine, Tongji University, Shanghai, China; d School of Medicine, Tongji University, Shanghai, China; e Department of Colorectal Disease, Tenth People’s Hospital, School of Medicine, Tongji University, Shanghai, China.

**Keywords:** cross-sectional study, diabetic complications, older adults, psychological distress, self-management, social-ecological support, type 2 diabetes mellitus

## Abstract

This study aims to explore the relationship among diabetes-related distress, social-ecological support, and self-management behavior in older adults with type 2 diabetes mellitus (T2DM) and chronic complications. This cross-sectional study included older adults with T2DM in Shanghai, China, between January and July 2022. The problem areas in diabetes scale (PAID), the chronic illness resource survey (CIRS), and the diabetes self-management behavior for older (DSMB-O) were employed. A total of 264 participants (157 [59.47%] males, aged 71.07 ± 6.47 years) were included; their T2DM duration ranged from 5 to 30 years, with an average of 11.19 ± 6.96 years. The DSMB-O scores were negatively correlated with the PAID scores and positively correlated with CIRS scores. The CIRS scores were negatively correlated with the PAID scores. CIRS had a positive direct effect on DSMB-O, and CIRS had an indirect effect on DSMB-O through PAID. CIRS had a total effect on DSMB-O through PAID. The mediating effect made up 28.89% of the total effect. In older adults with T2DM and chronic complications, chronic illness resources were correlated with diabetes-related distress and self-management behavior. Chronic illness resources had both a direct effect on self-management behavior and an indirect effect through diabetes-related distress.

## 1. Introduction

The global prevalence of diabetes is projected to reach 643 million by 2030 and 783.2 million by 2045.^[1]^ Type 2 diabetes mellitus (T2DM) constitutes more than 90% of all diabetes cases.^[1]^ The incidence of T2DM is increasing due to the aging population. In the United States in 2019, the prevalence of T2DM in individuals over 65 was 29.2%.^[2]^ By 2045, it is anticipated that the prevalence of T2DM in this age group will be 67.7 million in China.^[3]^

Patients with T2DM encounter chronic complications arising from inadequate blood glucose control, encompassing conditions such as blindness, kidney failure, cerebrovascular accidents, cardiovascular disease, amputation, and mortality.^[4]^ When stratified by age, a higher cumulative incidence of microvascular and macrovascular complications was observed in older patients versus younger patients.^[5]^ The escalating treatment expenses associated with the chronic complications of T2DM impose a substantial burden on the national health insurance system, constituting more than 80% of the total medical cost related to T2DM, particularly in the elderly population.^[6]^ Consequently, the chronic complications of T2DM in the elderly not only stand as the primary cause of death and disability but also significantly impact the quality of life and escalate the economic burden. Nonetheless, the chronic complications of T2DM are preventable and manageable even at the time of diabetes diagnosis.^[5]^ The median time to the onset of chronic complications of T2DM is 3 to 5 years, a critical period to implement preventive measures to impede the progression of chronic complications of T2DM.^[5]^ The principal cause of chronic complications of T2DM in the elderly is the neglect of self-care,^[7]^ encompassing aspects such as dietary habits, physical activity, medication adherence, emotional well-being, sleep, and medical attention.^[8]^ T2DM in the elderly with comprehensive knowledge and understanding of diabetes can adhere to self-management principles, resulting in improved glycemic control and better health outcomes.^[9]^ Cigolle et al^[10]^ demonstrated that older adults were more likely to face challenges in self-managing their diabetes and exhibited poorer health status.

The occurrence of chronic complications in T2DM in the elderly is not attributable to a singular factor but rather arises from a confluence of environmental and psychological elements, owing to its fundamentally psychological nature. As a theoretical framework, the health ecology^[11]^ model reveals that both individual and population health result from the intricate interplay of 5 factors. These include biological factors, exemplified by genetic predispositions, behavioral factors encompassing lifestyle choices and psychological aspects, interpersonal network factors involving family dynamics and social support, as well as factors associated with one’s working and living environment, and the prevailing policy environment. Beyond the domain of knowledge and literacy specific to T2DM in the elderly, numerous other factors emerge as pivotal contributors to the prevention of diabetic complications and the deceleration of disease progression, as viewed through the lens of the health ecology model. Several scholars characterize diabetes as a “complex, demanding, and often bewildering set of self-care directives,” a scenario wherein elderly patients with T2DM, who suffer from strictly controlled diet, regular activities, adhere to taking drugs, and its long-term complications,^[12]^ may experience feelings of frustration, anger, being overwhelmed, and/or discouragement. Psychological health emerges as a critical determinant influencing how individuals cope with and manage diabetes. Studies indicate that diabetes-related distress is pervasive, with a prevalence ranging from 18% to 35%. This type of diabetes-related distress refers to the adverse emotional reactions exhibited by patients when confronted with the demands of disease management, emotional burdens, and treatment.^[13]^ Older patients are more likely to suffer from emotional distress resulting from living with diabetes. The prevalence of moderate and severe distress among T2DM in elderly patients has been linked to suboptimal self-management practices and adverse impacts on disease management, leading to compromised glycemic control.^[14]^

Social support, defined as an individual’s perception of receiving assistance from diverse sources such as family members, friends, and other social contacts, plays a significant role in influencing health-related well-being.^[15]^ While it exerts a relatively weak predictive influence on diabetes-related distress,^[16]^ lower levels of diabetes-related distress are associated with support from physicians. The prospect of sharing thoughts and emotions with healthcare professionals or others is posited to mitigate negative emotions, fostering improved emotional adjustment.^[16]^ Yuichi Temma et al demonstrated that only 26.1% received high social support related to diabetes care from their families, friends, or neighbors, and found that only patients with high overall social support tended to have better glycemic control.^[17]^

As a remote factor in chronic complications of T2DM in the elderly, the influence of policy environment cannot be ignored. The policy level includes economic, social, cultural and related politics at the community, government, national and global levels. The variables selected in different studies mainly include medical insurance, pension insurance, and the utilization or satisfaction of community medical resources, etc.^[18]^

Nevertheless, numerous studies have substantiated the connections between diabetes-related distress, social-ecological support, and self-management behavior. However, the majority of these studies have concentrated solely on the bivariate relationships of variables, leaving the precise direction of these relationships ambiguous. The core concepts in our model encompass diabetes-related distress, social-ecological support, and self-management behavior. We posit that diabetes-related distress plays a mediating role in the correlation between social-ecological support and self-management behavior among older adults with T2DM and chronic complications.

## 2. Methods

### 2.1. Study design and participants

This cross-sectional study was carried out among older adults with T2DM in Shanghai, China, utilizing convenience sampling from January to July 2022. Inclusion criteria comprised individuals: aged ≥ 60 years, diagnosed with T2DM for a minimum of 5 years before the study, and presenting with at least 1 chronic complication of T2DM. Exclusion criteria included: critical illness, inability or refusal to provide informed consent, or severe psychiatric conditions.

Approval for this study was granted by the Committee of Research Ethics of the Hospital (No. SHSY-IEC-4.1/21-284/01). Participants provided informed consent before engaging in the study.

To safeguard the authenticity and precision of respondent answers, the survey was conducted anonymously. Study investigators, recruited from medical school students with a medical background, were employed to minimize bias in questionnaire responses.

The researcher directly contacted participants and arranged face-to-face interviews, allocating approximately 20 to 25 minutes per participant for data collection. Participants received no monetary incentives or reimbursements for their involvement in the study.

### 2.2. Questionnaire

The questionnaire encompassed 4 sections: demographic characteristics, the problem areas in diabetes scale (PAID), the chronic illness resource survey (CIRS), and the diabetes self-management behavior for older (DSMB-O).

For this study, a demographic questionnaire was devised, covering age, sex, race, education level, marital status, employment, living status, smoking, number of children, diabetic family history, income, sleeping habits, alcohol consumption, physical exercise, and duration of T2DM.

The PAID, employed to identify an individual’s diabetes-related emotional distress,^[19]^ is widely utilized and demonstrates satisfactory sensitivity (94%) and specificity (89%).^[19]^ Comprising 20 items distributed across 4 dimensions, the PAID assesses diabetes-related emotional problems (12 questions), treatment-related problems (3 questions), food-related problems (3 questions), and social support-related problems (2 questions).^[19]^ Respondents rate items on a 5-point scale (0–4, representing “Not a problem” through to “Serious problem”).^[19]^ The PAID has undergone translation into various languages, with established psychometric properties.^[20]^ A higher total score indicates a more pronounced issue with diabetes-related emotional distress.

The CIRS, developed by Glasgow et al,^[21]^ consists of 22 items and serves as a valuable metric for evaluating multifaceted, social-ecological support for the self-management of chronic illness. Its subscales encompass personal, family/friend, neighborhood, work, community/organization, health care team, and media resources.^[21]^ Each of the 22 items is assessed on a 5-point Likert scale ranging from 1 (not at all) to 5 (often). Subscale scores for the CIRS are computed by summing the scores for all items within the subscale and then dividing by the number of items in that subscale.^[22]^ The CIRS total score is derived by summing the subscale scores and dividing by 7.^[22]^ The Chinese version of the CIRS, featuring 27 items, exhibits commendable psychometric indicators.^[23]^ A higher total score on the CIRS indicates a greater abundance of resources.

The DSMB-O functions as a practical tool for evaluating diabetes self-management behaviors in older adults with type 2 diabetes.^[24]^ Comprising 14 items based on the 7 domains identified by the AADE, the DSMB-O includes items related to 6 domains that necessitate daily performance, rated on a 4-point Likert scale (0 = “Never” to 3 = “Always”). The “Reducing risks” domain encompasses 1 item derived from the evaluation of 5 subitems, designed to yield a dichotomous response (“Yes”/“No”). Scores for each of the 5 subitems range from 0 to 3, and the item score for the “Reducing risks” domain falls between 0 and 0.6. A higher total score on the DSMB-O indicates more effective diabetes self-management behaviors.

### 2.3. Statistical analysis

The minimum sample size for the Structural Equation Model (SEM) was computed to be 150.^[25]^ Factoring in a 15% dropout rate, a recruitment goal of at least 173 patients with T2DM was established. Statistical analyses were executed using SPSS 23.0 (IBM, Armonk, NY), while the SEM utilized AMOS 26.0 (IBM). Continuous data adhering to a normal distribution were presented as mean ± standard deviation (SD) and compared using the Student *t* test or one-way ANOVA with the LSD post hoc test. Categorical data were expressed as n (%) and subjected to comparison via the chi-square test. Pearson correlation test was employed to explore relationships among DSMB-O, PAID, and CIRS. A 2-sided *P* < .05 was considered indicative of a significant difference.

The SEM was deemed apt for constructing a model elucidating relationships among characteristics based on the variance/covariance matrix, utilizing maximum likelihood estimation. Evaluation of the hypothesized model involved multiple goodness-of-fit criteria: *χ*^2^/df < 3, goodness-of-fit index (GFI) > 0.9, comparative fit index (CFI) > 0.9, Tucker–Lewis index (TLI) > 0.9, root mean square error of approximation (RMSEA) index < 0.08, and standardized root mean square residual (SRMR) < 0.08.^[26]^

## 3. Results

Out of the 272 enrolled patients, 264 (157 males [59.47%], aged 71.07 ± 6.47 [range, 60–96] years) provided valid questionnaires, yielding an effective recovery rate of 97.06%. The duration of T2DM ranged from 5 to 30 years, with an average of 11.19 ± 6.96 years. Around <21% of patients lived alone, and over 90% of participants had an education level of primary school or below. About 75.80% of patients primarily relied on urban medical insurance for their medical coverage (Table 1).

**Table 1:** Sociodemographic, PAID, CIRS, and DSMB-O in elderly.

Characteristics	N (%)	DSMB-O		CIRS		PAID	
		Mean ± SD	*P*	Mean ± SD	*P*	Mean ± SD	*P*
Age (yr)			.43		.02		.78
60–69	84 (31.82)	0.85 ± 0.30		2.27 ± 0.66		2.64 ± 0.58	
70–75	61 (23.11)	0.93 ± 0.39		2.58 ± 0.82		2.61 ± 0.68	
≥76	119 (45.08)	0.86 ± 0.39		2.53 ± 0.79		2.58 ± 0.71	
Sex			.12		.18		.04
Female	107 (40.53)	0.83 ± 0.34		2.38 ± 0.68		2.71 ± 0.62	
Male	157 (59.47)	0.90 ± 0.38		2.51 ± 0.82		2.54 ± 0.68	
Education			.43		.27		.61
Primary school or below	241 (91.29)	0.87 ± 0.36		2.44 ± 0.77		2.61 ± 0.66	
Secondary school or above	23 (8.71)	0.93 ± 0.38		2.63 ± 0.70		2.54 ± 0.64	
Marital status			.09		<.01		.08
Single*	52 (19.70)	0.80 ± 0.32		2.15 ± 0.54		2.73 ± 0.53	
Married	212 (80.30)	0.89 ± 0.37		2.53 ± 0.79		2.58 ± 0.69	
Employment			.99		.66		.73
None or retired	232 (87.88)	0.88 ± 0.37		2.47 ± 0.77		2.61 ± 0.67	
Employed	32 (12.12)	0.88 ± 0.30		2.40 ± 0.73		2.57 ± 0.58	
Living status			.78		.08		.15
Living alone	55 (20.83)	0.89 ± 0.38		2.32 ± 0.64		2.50 ± 0.57	
Living with family/others	209 (79.17)	0.87 ± 0.36		2.50 ± 0.79		2.63 ± 0.68	
Number of children			.59		.42		.17
0	25 (9.47)	0.87 ± 0.32		2.27 ± 0.53		2.59 ± 0.44	
1	156 (59.09)	0.89 ± 0.39		2.48 ± 0.81		2.64 ± 0.69	
≥2	83 (31.44)	0.84 ± 0.33		2.47 ± 0.74		2.50 ± 0.69	
Diabetic family history			.52		.78		.26
No	167 (63.26)	0.87 ± 0.38		2.47 ± 0.78		2.57 ± 0.68	
Yes	97 (36.74)	0.86 ± 0.33		2.44 ± 0.75		2.67 ± 0.63	
Income (RMB)			.04		.12		<.01
<3000	32 (12.12)	0.74 ± 0.29		2.41 ± 0.73		2.39 ± 0.68	
3000–6000	116 (43.94)	0.92 ± 0.40		2.57 ± 0.74		2.51 ± 0.66	
≥6000	116 (43.94)	0.86 ± 0.33		2.36 ± 0.79		2.77 ± 0.63	
Sleeping			<.01		.33		.01
Bad	107 (40.53)	0.81 ± 0.31		2.41 ± 0.67		2.76 ± 0.59	
Generally good	128 (48.48)	0.88 ± 0.36		2.46 ± 0.80		2.51 ± 0.67	
Great	29 (10.98)	1.08 ± 0.49		2.65 ± 0.95		2.48 ± 0.80	
Smoking			.18		.16		<.01
No	197 (74.62)	0.90 ± 0.37		2.51 ± 0.78		2.52 ± 0.67	
Yes	67 (25.38)	0.83 ± 0.33		2.36 ± 0.70		2.82 ± 0.59	
Alcohol consumption			.44		.48		.30
No	219 (82.95)	0.87 ± 0.37		2.47 ± 0.76		2.63 ± 0.67	
Yes	45 (17.05)	0.91 ± 0.33		2.39 ± 0.79		2.51 ± 0.60	
Physical exercise			.04		.02		.08
No	140 (53.03)	0.83 ± 0.36		2.36 ± 0.76		2.67 ± 0.66	
Yes	124 (46.97)	0.93 ± 0.37		2.58 ± 0.75		2.53 ± 0.66	
Duration of T2DM (yr)			.65		.58		.05
5–9	138 (52.27)	0.88 ± 0.37		2.41 ± 0.84		2.52 ± 0.70	
10–19	74 (28.03)	0.90 ± 0.35		2.50 ± 0.71		2.64 ± 0.61	
≥20	52 (19.70)	0.84 ± 0.37		2.52 ± 0.60		2.78 ± 0.58	
Medical insurance			.04		.01		.53
Self-pay	12 (4.50)	0.71 ± 0.25		2.30 ± 0.45		2.60 ± 0.54	
Urban medical insurance	200 (75.80)	0.91 ± 0.38		2.70 ± 0.69		2.63 ± 0.65	
New rural cooperative medical care	51 (19.30)	0.79 ± 0.29		2.44 ± 0.50		2.55 ± 0.72	
Others	1 (0.40)	0.50 ± 0.00		1.61 ± 0.00		1.75 ± 0.00	

CIRS = chronic illness resource survey, DSMB-O = diabetes self-management behavior for older, PAID = problem areas in diabetes scale, SD = standard deviation, T2DM = type 2 diabetes mellitus.

* Never married, divorced, separated, and widowed.

Table 2 displays the total and subscale scores of DSMB-O, CIRS, and PAID scores for all participants. The mean DSMB-O score was 0.88 ± 0.36. Higher income (*P* = .004), good sleep quality (*P* < .01), physical exercise (*P* = .04), and urban medical insurance (*P* = .04) were associated with higher DSMB-O scores. The mean CIRS score was 2.46 ± 0.76. Older age (*P* = .02), being married (*P* < .01), physical exercise (*P* = .02), and urban medical insurance (*P* = .01) were associated with higher CIRS scores. The mean PAID scores were 2.61 ± 0.66. Female sex (*P* = .04), higher income (*P* < .01), poor sleep quality (*P* = .01), smoking (*P* < .01), and longer T2DM duration (*P* = .05) were associated with higher PAID scores (Table 1).

**Table 2:** Descriptive statistics of the study variables.

Characteristics	Mean ± SD
Problem areas in diabetes scale scores	2.61 ± 0.66
Emotional problems	2.66 ± 0.81
Food-related problems	2.60 ± 0.66
Treatment-related problems	2.61 ± 0.69
Social support-related problems	2.64 ± 0.82
Chronic illness resource survey	2.46 ± 0.76
Healthcare team	2.60 ± 0.89
Family/friend	2.32 ± 1.01
Personal	2.57 ± 0.82
Neighborhood	2.34 ± 0.97
Media resources	2.55 ± 1.09
Community/organization	2.33 ± 0.98
Work	0.30 ± 0.86
Diabetes self-management behavior for older	0.88 ± 0.36
Active exercise	1.14 ± 0.96
Eating a healthy diet	1.49 ± 0.70
Medication	1.36 ± 0.96
Blood sugar monitoring	1.55 ± 0.91
Deal with problems	1.26 ± 0.92
Actively respond to problems	1.11 ± 0.97
Reducing risks	0.12 ± 0.17

SD = standard deviation.

Pearson correlation analysis reveals that DSMB-O scores were correlated with PAID scores (*r* = −0.46, *P* < .001) and CIRS scores (*r* = 0.55, *P* < .001). CIRS scores were correlated with PAID scores (*r* = −0.50, *P* < .001) (Table 3).

**Table 3:** Correlations of PAID, CIRS, and DSMB-O for the elderly with chronic complications of T2DM.

	PAID	CIRS	DSMB-O
PAID	1.00		
CIRS	−0.50*	1.00	
DSMB-O	−0.45*	0.54*	1.00

CIRS = chronic illness resource survey, DSMB-O = diabetes self-management behavior for older, PAID = problem areas in diabetes scale, T2DM = type 2 diabetes mellitus.

* *P* < .001.

**Figure 1.**
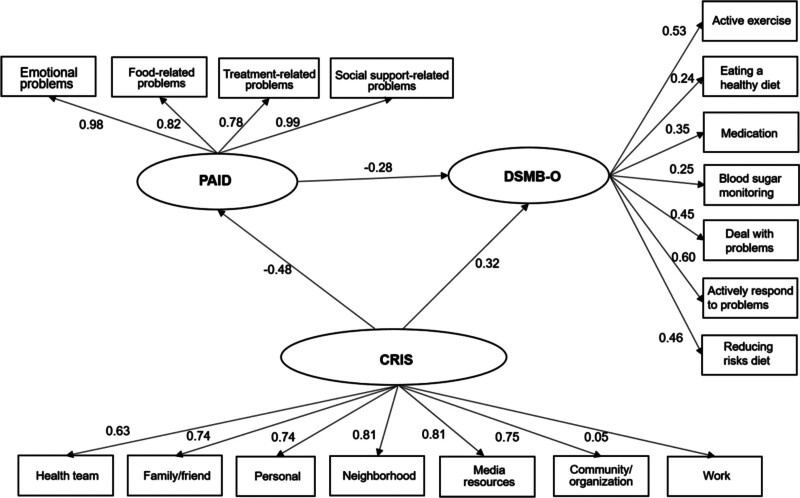
Research framework and hypothesis model. CRIS = chronic illness resource survey, DSMB-O = diabetes self-management behavior for older, PAID = problem areas in diabetes scale.

Building upon the correlation analysis above and previous research findings, a Structural Equation Model (SEM) was formulated to investigate the relationship among PAID, CIRS, and DSMB-O in older adults with chronic complications of T2DM. The influence of CIRS on DSMB-O in older adults with chronic complications of T2DM encompassed both a direct path and an indirect path: CIRS exhibited a direct effect on DSMB-O (0.32), and CIRS exerted an indirect effect on DSMB-O through PAID (−0.48 × −0.28 = 0.13). The cumulative effect of CIRS on DSMB-O through PAID was calculated as (0.13 + 0.32 = 0.45), with the mediating effect constituting 28.89% (0.13/0.45) of the total effect. The final fit indices of the model were *χ*^2^/df = 1.757, GFI = 0.934, CFI = 0.971, TLI = 0.950, RMSEA = 0.062, and SRMR = 0.074 (Fig. 1).

## 4. Discussion

This study demonstrated a correlation between DSMB-O scores, PAID scores, and CIRS scores in older adults with T2DM and chronic complications. The CIRS scores were inversely correlated with the PAID scores (*r* = −0.50). CIRS exhibited both a direct impact on DSMB-O and an indirect effect on DSMB-O through PAID. Significant associations were uncovered among diabetes-related distress, social-ecological support, and self-management behavior in this population.

The investigation revealed that older adults with T2DM and chronic complications exhibited low self-management behavior. As reported by Qi et al,^[27]^ T2DM in the elderly with complications could lead to a diminished quality of life, a consequence directly influenced by inadequate self-management behaviors. Self-management involves actively participating in self-care activities to enhance behaviors and well-being. Managing T2DM encompasses lifestyle adjustments (dietary management, planned physical activities, blood glucose monitoring, taking diabetes medicines, stress management), handling episodes of illness, monitoring blood glucose levels,^[28]^ and periodic health reviews, so as to delay the development of T2DM with chronic complications. In this study, the “risk reduction” dimension scored the lowest, signifying that older adults with T2DM and chronic complications should undergo regular hospital visits for physical examinations. Armstrong et al^[29]^ emphasized the critical importance of early lesion recognition in patients with a history of diabetic foot ulcers to reduce the risk of complications. The elderly patient self-management programs play a crucial role in averting acute complications and mitigating the risk of long-term complications.

In this study, the level of social-ecological support fell below satisfaction, with a score of 2.46 ± 0.76, indicating a value below 3 (the intermediate point). These findings align with Zhang et al’s work.^[30]^ Notably, the ‘healthcare team’ subdomain obtained the highest score among all subdomains, underscoring the pivotal role of medical staff support in providing direct guidance for enhancing patients” self-care capabilities.^[31]^ Conversely, the “family/friend” subdomain scored the lowest. Family support involves fostering communication, aiding in accessing health services, reminding elderly patients to regularly take prescribed blood sugar control medications, organizing their diet, and monitoring blood sugar levels.^[32]^ The caregiver proficiency in diabetes knowledge and appropriate supportive behaviors can significantly influence the quality of life for older adults with T2DM. Practical support from family and friends in facilitating adherence to medication, diet, and exercise is integral to achieving optimal glycemic control.^[33]^ Furthermore, emotional support from family members, who express concern for the patients’ condition and offer encouragement and care, plays a significant role. Thus, healthcare providers should prioritize motivating and empowering family caregivers to be more attentive to the elderly with diabetes.^[34]^

In this study, the participant diabetes-related distress score (2.61 ± 0.66) exceeded 2, the intermediate point, consistent with literature findings.^[13]^ The rank of dimension scores from high to low was emotional problems, social support-related problems, treatment-related problems, and food-related problems. A previous study suggested an association between diabetes-related distress and insulin therapy and total sleep time. This phenomenon may be attributed, first, to the necessity for patients to modify their diet, sleep, and lifestyle factors to maintain stable blood glucose, potentially exacerbating negative emotions.^[35]^ Second, the prolonged and intricate nature of multidisciplinary collaborative treatment for chronic complications of diabetes may contribute to distress. Recent evidence indicates that psychological factors such as depressive symptoms or perceived stress elevate the risk of microvascular and macrovascular complications associated with T2DM.^[36]^ Consequently, it is imperative to focus on the emotional well-being of older adults with T2DM and chronic complications. Psychological interventions, including cognitive-behavioral and psychodynamic supportive therapies, have demonstrated efficacy in improving depression severity and promoting depression remission both at the end of the study period and up to 6 months later.^[36]^

Based on Pearson correlation analyses, social-ecological support exhibited a positive correlation with self-management behaviors, consistent with findings from a prior study.^[37]^ Furthermore, an inverse correlation between emotional distress and self-management behaviors was identified, aligning with the observations made by Kattika et al^[14]^ The Structural Equation Model (SEM) illustrated that social-ecological support had an indirect influence on self-management behaviors through diabetes-related distress in older adults with T2DM and chronic complications, elucidating the explanatory role of these 2 variables (social-ecological support and diabetes-related distress) in the context of diabetes self-management behavior.

A recent study has underscored the crucial role of social-ecological support in facilitating successful self-management among patients with T2DM.^[38]^ Comprehensive support from the healthcare team, family/friends, media resources, and the community is essential in fostering self-management behaviors. This emphasizes the positive association between social-ecological support and self-management behaviors.^[39]^ Moreover, the act of sharing thoughts and feelings with healthcare professionals or others is shown to mitigate negative emotions and contribute to improved emotional adjustment for T2DM.^[40]^ Chronic illness resources, encompassing various tools for self-management in individuals with chronic conditions, serve to alleviate diabetes-related distress and sustain effective self-management behaviors.^[30]^ Importantly, social-ecological support influences diabetes-related distress, subsequently impacting patient self-management behaviors. This study suggests that diabetes-related distress serves as a partial mediator in the relationship between social-ecological support and self-management behaviors. Medical staff can employ diverse strategies to enhance self-management behaviors in older adults with T2DM and chronic complications. This can be achieved by bolstering social support through judicious resource utilization or by addressing diabetes-related distress through a cognitive psychological approach, particularly in elderly diabetes. Consequently, an intervention focusing on enhancing both social-ecological support and mitigating diabetes-related distress may yield improved self-management behaviors of elderly diabetes with chronic complications.

Several limitations warrant consideration in this study. The cross-sectional design necessitated the use of convenience sampling, potentially introducing sampling bias and limiting the generalizability of the findings to populations in other geographic regions. Additionally, reliance on self-report measures introduces the possibility of response bias, wherein respondent answers may deviate due to psychological or personality predispositions.^[30]^ Moreover, social desirability bias could be present, as some respondents may alter their true intentions to create a positive impression or protect themselves, aligning with social expectations. Despite these limitations, the use of self-report measures remains one of the most practical methods for data collection.^[30]^

In conclusion, within the cohort of older adults with T2DM and chronic complications, social-ecological support exhibited correlations with diabetes-related distress and self-management behavior. Social-ecological support exerted both a direct impact on self-management behavior and an indirect effect through diabetes-related distress. This study disclosed inadequacies in social-ecological support, diabetes-related distress, and self-management behaviors among older adults with T2DM and chronic complications in China. Notably, social-ecological support and diabetes-related distress exhibited positive correlations with self-management behaviors. Furthermore, diabetes-related distress was identified as a partial mediator in the relationship between social-ecological support and self-management behaviors.

## Acknowledgments

The authors express gratitude for the invaluable contributions of the participants and their families.

## Author contributions

**Conceptualization:** Xiao Sun, Yan Shi.

**Formal analysis:** Xiao Sun, Yan Shi, Rongrong Zhou.

**Investigation:** Xue Wang.

**Methodology:** Xue Wang, Rongrong Zhou.

**Resources:** Wei Deng.

**Supervision:** Wei Deng.

**Writing—original draft:** Xiao Sun.
